# Allergic conjunctivitis increases the likelihood of undergoing eyelid incision surgery in pediatric and adolescent patients

**DOI:** 10.1038/s41598-022-09668-x

**Published:** 2022-04-06

**Authors:** Li Lyung Wang, Ji-Won Kwon, Ju-Yeun Lee

**Affiliations:** 1 Department of Ophthalmology, Myongji Hospital, Hanyang University College of Medicine, Goyang, South Korea; 2 Department of Preventive Medicine, Seoul National University College of Medicine, Seoul, South Korea; 3 Integrated Major in Innovative Medical Science, Seoul National University College of Medicine, Seoul, South Korea

**Keywords:** Conjunctival diseases, Eyelid diseases

## Abstract

The global prevalence of allergic diseases has increased dramatically in recent decades. From a global health perspective, they have been considered as a major chronic disease, and the related social burden has also been increasing worldwide. In line with this trend, we investigated the likelihood of undergoing incision surgery for eyelid inflammatory masses in pediatric and adolescent patients with allergic conjunctivitis (AC). The prevalence of AC and incision surgery showed a similar pattern of bimodal peaks during the spring and autumn of South Korea, reflecting the peak allergic seasons. The likelihood of undergoing incision surgery in patients with AC was 4.27 times higher than that of patients without AC and the likelihood of undergoing incision surgery was higher in every age group in the AC cohort than in the non-AC cohort. There was no significant difference between age groups and sexes. In the AC cohort for sub-analysis, the likelihood of undergoing incision surgery was 2.34 times higher in patients who used steroids than those who did not. These findings can suggest that proper management and education can be required for the likelihood of undergoing incision surgery due to eyelid inflammation mass in pediatric and adolescent patients with AC. Furthermore, greater attention should be paid to patients using steroids due to severe AC as they are more likely to undergo incision surgery.

## Introduction

Hordeolum and chalazion are examples of localized sudden onset swelling of the eyelids. Chalazion generally results from blockage of the meibomian gland in the eyelid, whereas hordeolum is caused by infection. Both conditions are characterized by eyelid swelling, hyperemia, and pain. With early clinical diagnosis and proper management, both conditions can be treated immediately. Moreover, when the lesions become large, unsightly, or persist over time, an incision may be needed to hasten the resolution.

Along with several underlying medical conditions, such as blepharitis and atopic or seborrheic skin conditions, it is widely known that infectious conjunctivitis can affect the development of the hordeolum or chalazion. In addition to infectious conjunctivitis, AC promotes an immunopathological system, and frequent rubbing, which accelerates the immunologic process, can lead to secondary eyelid inflammation. Therefore, patients with AC may have a higher likelihood of undergoing incision surgery with the hordeolum and chalazion than those without AC.

In pediatric patients, there are several limitations in managing these medical conditions due to their poor cooperation and refusal to use proper medications or hot compression. Moreover, when incision surgery is required, it is inevitable to use general anesthetics for a safe and efficient treatment. Particularly in younger patients, frequent contact cannot be controlled, which can affect eyelid inflammation. Thus, managing chalazion or hordeolum could become a burden for caregivers. Such clinical experiences due to frequent incision surgeries motivated us to assess its risk in allergic conjunctivitis (AC). Although it can be easily diagnosed in clinics, few studies have illuminated this relationship. Therefore, we aimed to evaluate the likelihood of undergoing incision surgery for eyelid inflammation, such as chalazion or hordeolum, in pediatric and adolescent patients with AC using sample data obtained from a nationwide population-based cohort.

## Methods

### Data sources

We accessed a one million population-based sample data recorded by the Health Insurance Review and Assessment (HIRA) service of South Korea in 2018. This claim database holds all health care information from both inpatients and outpatients using codes from the Korean Standard Classification of Diseases (KCD), 7th revision with a few changes related specifically to Korea based on the International Classification of Diseases (ICD), 10th revision. The novelty, as well as detailed information regarding the HIRA database, has been provided in our previous study^1,2^.

The current nationwide cohort study complied with the Declaration of Helsinki and was approved by the Institutional Review Board of the Myongji Hospital, South Korea (IRB No. 2020-10-040). The requirement for informed consent was waived by the Institutional Review Board of the Myongji Hospital, South Korea due to the retrospective design of the study and the anonymous nature of the data.

### Participants and sample selection

All patients who had possible AC (registration code H101–H103) during the one-year study period and had a continuous enrollment of two independent visits were included in our estimates. All pediatric participants who were not diagnosed with AC at the same time were considered as controls and included in the non-AC cohort. All enrollees with these registration codes were confirmed and registered by an ophthalmologist to ensure AC diagnosis. Furthermore, to exclude other types of conjunctivitis such as atopic or vernal keratoconjunctivitis, those who had a history of keratoconjunctivitis (H1620), keratoconjunctivitis sicca (H1621), neurotrophic keratoconjunctivitis (H1622), phlyctenular keratoconjunctivitis (H1624), vernal keratoconjunctivitis (H1625), and any other or unspecified keratoconjunctivitis (H1628-9) were excluded. Participants with AC who were finally enrolled in the study were referred to a combination of diagnosis code and simultaneous prescription of eye drops for AC.

To help exclude conditions that can affect the development of hordeolum, those who had any history of acne (L700-719), blepharitis (H011-019), atopic/seborrheic skin disease (L208-219), leishmaniasis (B551, B559, Q909), or any other infectious conditions, including herpes zoster (B023), tuberculosis (A185), diphtheria (A368), meningococcus (A398), gonococcus (A543), trachoma (A71) chlamydia (A74, H131), measles (B05), acanthamoebiasis (B60), and any other bacterial/viral conjunctivitis (B300-309) were excluded.

For the prevalence estimates, the date of the earliest claim along with their registration code was defined as the index date, and the patient was considered as an incident case for that year. Moreover, most of the patients in this database were Asian. All pediatric participants who were not diagnosed with AC simultaneously were considered as controls and included in the non-AC cohort. Medical records refer to all billing data that include the diagnosis code, treatment, prescribed drug, and visit date generated every time a patient visits a medical clinic.

### Analyses

#### Prevalence

We categorized the pediatric and adolescent patients into eight groups based on four strata of age as well as sex. Since AC is generally diagnosed based on patients’ symptoms and the clinical findings obtained by slit-lamp microscopy, patients under 3 years of age were excluded considering the degree of cooperation of patients who can undergo slit-lamp examination. The pediatric and adolescent patients were classified into strata of age between 3–5, 6–9, 10–12, and 13–19 which had been established by HIRA system. Those diagnosed with AC within a year of the study were also included in the prevalence estimates. The monthly distribution of AC diagnosis, as well as incision surgeries, were estimated and noted.

#### Establishing cohort and ascertainment of outcome

The current cohort data was established to evaluate the likelihood of undergoing incision surgery in patients with AC. Based on the exposure of AC diagnosis, the AC cohort and non-AC cohort were established for the final analysis. All incidental AC cases were enrolled in the AC cohort, and participants who met clinicians with medical records due to diseases other than AC were obtained from the same period and included in the non-AC cohort, and exclusive diseases were excluded. To establish a one-year cohort and remove potential preexisting cases of surgery, the first 2 months (January and February 2018) were set as the wash-out period. The wash-out period was set to ensure that there were no identified cases of incision surgery before the diagnosis of AC.

To identify possible episodes of incision surgery, the patients were linked to the outpatient and inpatient records using an encrypted personal identification key. In the current cohort, patients who newly underwent incision surgery (S5400, S5250) for hordeolum or chalazion after the index date of AC diagnosis were determined as incident cases. Since the surgical codes were only used for the incision for chalazion or hordeolum, there would be no other reasons for incision surgery in this study setting. Moreover, we also censored the follow-up time at the end of this study for anonymity. (December 31, 2018).

### Statistics

Data handling and statistical analyses were performed by an independent data analyst (J. L.) specially trained by the HIRA institute for their 2017 HIRA big data. A comparison of continuous variables between the groups was performed using the paired t-test, and a comparison of the proportion of each variable between the groups was analyzed using the Chi-squared test. The stratified log-rank test was used to compare the incidence rates of surgery between the AC and non-AC groups. Cox proportional hazards regression with a cluster effect was used to compute the adjusted hazard ratios for the two groups. Covariates in the multivariable Cox proportional hazards regression to compute the adjusted hazard ratios included age group, sex, and allergic rhinitis. The event refers to the first index date of incision surgery. The time-to-event in the statistical model refers to the interval between the first index date of AC diagnosis and incision surgery. Otherwise, in cases of no event, the endpoint of follow-up was December 31, 2018. For sub-analysis, we selected the cohort data of the AC group only. In the AC group, the likelihood of undergoing incision surgery was computed using Cox proportional hazards regression with covariates including age group, sex, use of steroids at the time of AC diagnosis, and presence of allergic rhinitis. Because steroid eye drops are primarily used in severe AC, we additionally obtained the use of steroid eye drops at the diagnosis of AC, which can indirectly reflect the severity of AC. A confidence level of 95% was used for this analysis, and all results are presented as mean ± standard deviation (SD). The period prevalence was estimated as the number of patients identified with AC divided by the total population into the sample cohort data. The 95% CIs of the prevalence was estimated using Poisson distribution. *P*-value less than 0.05 indicated statistical significance. SAS Enterprise Guide version 6.1 software (SAS Inc., Cary, NC, USA) was used for all analyses.

## Results

### Demographics

In our study, a total of 66,657 patients comprising 33,389 males and 33,268 females were diagnosed with simple AC code between January 2018 and December 2018. After excluding cases that did not have a prescription for eye drops and with a prior history of exclusive diseases, 15,703 patients, including 8313 males and 7390 females, were finally diagnosed with AC.

### Prevalence of allergic conjunctivitis

**Figure 1:**
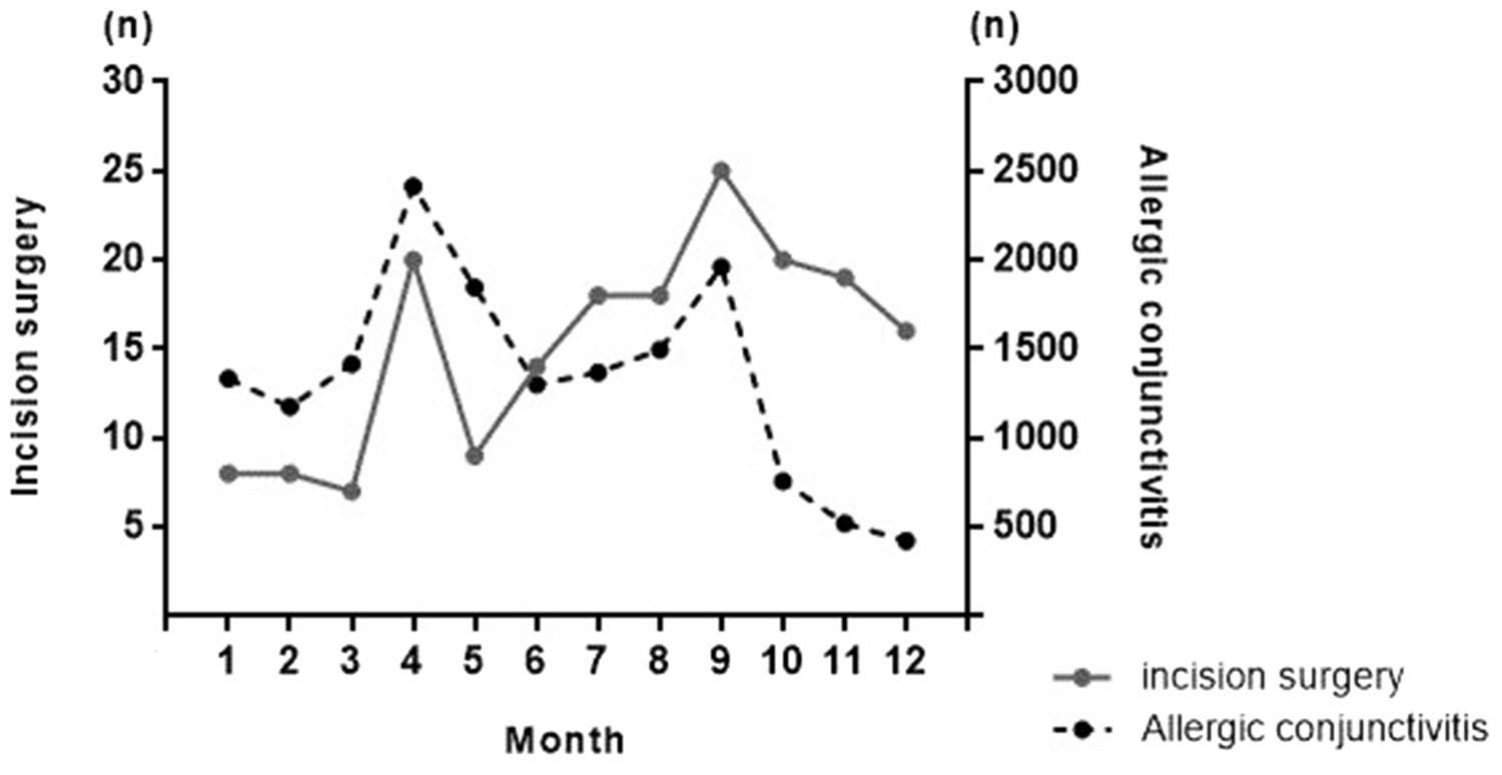
Monthly distribution of allergic conjunctivitis (AC) and incision surgery for chalazion and hordeolum in a pediatric and adolescent population using the population-based sample data, South Korea.

The prevalence of AC in the study population, aged between 3 and 19 during the one-year study period, was 940.63 (95% CI, 904.62–976.64) per 10,000 people. In males, it was 951.76 (95% CI, 882.53–1020.99) per 10,000 people and in females, it was 928.41 (95% CI, 853.39–1003.44) per 10,000 people. The mean prevalence was higher in males than in females in age groups before 13 years of age. Overall, the male-to-female prevalence ratio was 1.03. The detailed results are presented in Table 1. The monthly distribution of AC diagnosis and incision surgery is shown in Fig. 1.

**Table 1:** Number of patients with allergic conjunctivitis and estimated prevalence rate (per 10,000 people) of allergic conjunctivitis in the South Korean Population based sample data of 2018.

Age group (years)	Total		Males		Females		Male–Female ratio
	No.	Prevalence (95% CI)*	No.	Prevalence (95% CI)*	No.	Prevalence (95% CI)*	
3–5	3706	1713.9 (1338.7–2089.2)	2077	1878.3 (1110.1–2646.5)	1629	1541.9 (813.4–2270.4)	1.22
6–9	5387	1529.7 (1312.0–1747.3)	2970	1645.1 (1204.7–2085.4)	2417	1408.3 (979.7–1836.8)	1.17
10–12	2792	974.6 (761.0–1188.1)	1511	1025.9 (599.7–1452.2)	1281	920.2 (493.1–1347.3)	1.11
13–19	3818	468.7 (416.6–520.8)	1755	403.4 (312.9–493.9)	2063	543.6 (423.2–664.1)	0.74
Total	15,703	940.6 (904.6–976.6)	8313	951.8 (882.5–1021.0)	7390	928.4 (853.4–1003.4)	1.03

*CI confidence interval.

### Predictors of incision surgery in the study group

In the established cohort, a total of 15,694 patients including 8309 males and 7385 females were enrolled in the AC cohort. The non-AC cohort consisted of 151,213 participants (79,022 males and 72,191 females) (Table 2). The median age of the cohort was 11.8 years. In the AC group, 21.3% of patients used topical antibiotics, and 38.1% of patients used topical corticosteroids at the time of AC diagnosis before incision surgery. Within a year, incident cases of incision surgery were performed in 39 (0.25%) patients in the AC group and 143 (0.09%) in the non-AC group. The surgery incidence rate within a year was 43.69 of 10,000 person-years for the AC group, while it was 11.71 of 10,000 person-years for the non-AC group. The total cumulative incidence rate was significantly higher in the AC group than in the non-AC group (*P* < 0.001). In a multivariate Cox regression analysis, the likelihood of undergoing incision surgery among all patients with AC was 4.27 times higher than that of the non-AC group (95% CI 2.95–6.18). The likelihood of undergoing incision surgery stratified by age group and sex is shown in Table 3. The mean follow-up period was 286.5 ± 83.1 days. The interval between AC diagnosis and incident surgery was 75.4 ± 83.5 days (median, 76 days).

**Table 2:** Demographic of allergic conjunctivitis and non-allergic conjunctivitis groups in the established short-term cohort data using population-based cohort sample data in South Korea.

Variable	AC group (n = 15,694)	Non-AC group (n = 151,213)	*P* value
Age group (n, %)			< 0.001
3–5	3703 (23.6)	17,916 (11.9)	
6–9	5389 (34.3)	29,827 (19.7)	
10–12	2791 (17.8)	25,854 (17.1)	
13–19	3811 (24.3)	77,616 (51.3)	
Sex (n, %)			0.103
Male	8309 (52.9)	79,022 (52.3)	
Female	7385 (47.1)	72,191 (47.7)	
Allergic rhinitis	13,834 (88.1)	103,546 (68.5)	< 0.001
Incision surgery (n, %)	39 (0.25)	143 (0.09)	< 0.001
Hordeolum	18 (46.2)	75 (52.5)	
Chalazion	21 (53.8)	68 (47.5)	
Follow up (days)	208 ± 90	295 ± 78	< 0.001

*AC* allergic conjunctivitis.

**Table 3:** Likelihood of undergoing incision surgery in AC group compared with non-AC group (reference group) in each subgroup analysis.

	Case (N, %)	HR (95% CI)^a^	p value
**Total population**			< 0.001
No AC	416 (0.07)	1.00	
AC	321 (0.25)	4.27 (2.95–6.18)	

Age groups	Case (N, %)	HR (95% CI)^b^	p value
**3–5 years**			0.016
No AC	46 (0.03)	1.00	
AC	80 (0.14)	4.07 (1.29–12.82)	
**6–9 years**			< 0.001
No AC	95 (0.07)	1.00	
AC	153 (0.33)	4.81 (2.53–9.13)	
**10–12 years**			0.002
No AC	47 (0.06)	1.00	
AC	35 (0.24)	4.68 (1.81–12.11)	
**13–19 years**			0.001
No AC	228 (0.13)	1.00	
AC	53 (0.37)	3.54 (1.93–6.50)	

*Sex*	Case (N, %)	HR (95% CI)^c^	p value
**Female**			< 0.001
No AC	254 (0.09)	1.00	
AC	126 (0.23)	4.41 (2.72–7.16)	
**Male**			< 0.001
No AC	162 (0.05)	1.00	
AC	192 (0.26)	3.98 (2.25–7.05)	

*AC* allergic conjunctivitis, *HR* hazard ratio, *CI* confidence interval.

^a^Adjusted for sex, age, and presence of allergic rhinitis.

^b^Adjusted for sex, and presence of allergic rhinitis.

^c^Adjusted for age, and presence of allergic rhinitis.

### Sub-analysis in the study group

**Figure 2:**
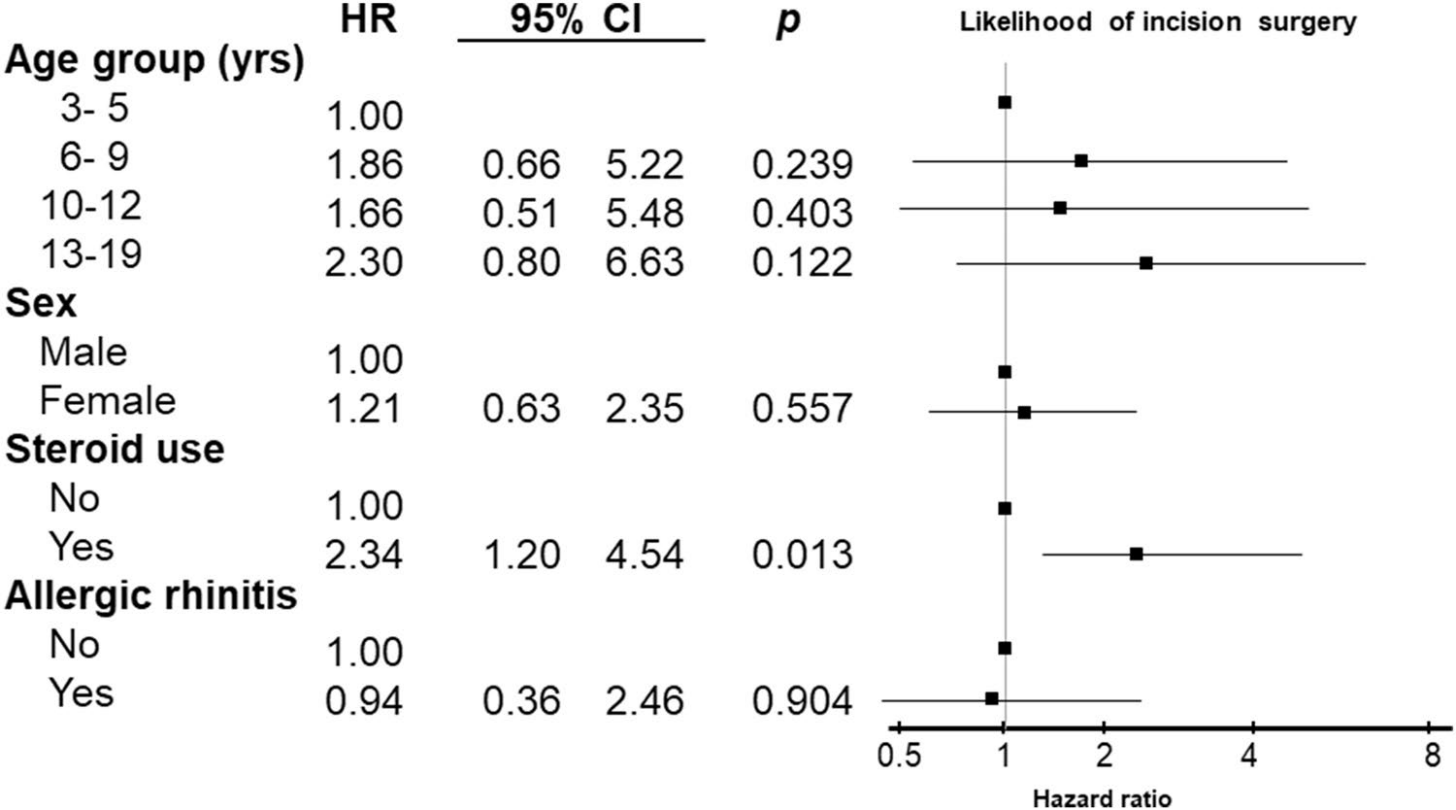
Predictors of incision surgery in the pediatric and adolescent population with allergic conjunctivitis (AC).

In AC cohort, estimates for the likelihood of undergoing incision surgery by age group, sex, use of steroids, and allergic rhinitis is shown in Fig. 2. The likelihood of undergoing incision surgery was 2.34 times higher in patients who used steroids at the diagnosis of AC than those who did not. (95% CI 1.20– 4.54). There is no difference in likelihood of undergoing incision surgery between age group, sex and the presence of allergic rhinitis at the diagnosis of AC (all *p* > 0.05).

## Discussion

The global prevalence of allergic diseases has increased dramatically in recent decades. From a global health perspective, they have been considered as a major chronic disease, and the related social burden has also been increasing worldwide. Based on a systematic review that reported temporal trends of allergic diseases in Korea, patterns of allergic rhinitis, as well as AC prevalence over time, has increased from 1.7 to 13.3%, while the authors speculate that the peak is yet to come for allergy epidemics^3^. In addition, to environmental factors such as urbanization and industrialization, the significant effect of climatological fine dust leading to dusty air pollution may accelerate the risk of allergic diseases in the general population^4^. Therefore, in situations where the influence of fine dust is quite large, the prevention and management of allergic diseases, such as AC and its complications, has become increasingly important in Korea. In line with this global trend, we investigated the recent prevalence of AC and the likelihood of undergoing incision surgery to treat a secondary eyelid inflammation mass.

In the current study, approximately 10% of the sample population had AC. In a recent study from India, the prevalence of AC was reported to be 12.22% among those aged between 5 and 15 years^5^. In another study, AC alone was found in 6–30% of the general population and up to 30% of children alone^6^. From Japan, there have been reports of AC prevalence in an estimated 14–27% of the pediatric population^7^. The relatively lower prevalence of AC in our study may be due to the use of fine definitions, such as prescription and other exclusion criteria. In addition, using data from a population-based sample data rather than the entire population with varying ethnicity and different age strata may also have yielded the difference. However, the prevalent age groups and differences in sex were consistent with the results of previous studies. Interestingly, the prevalence of AC and incision surgery showed a similar pattern of bimodal peaks during the spring and autumn of South Korea, reflecting the peak allergic seasons. These results suggest a possible link between AC and incision surgery for the treatment of eyelid inflammatory masses.

We found a higher likelihood of undergoing incision surgery in the study population with AC. It is widely known that conjunctivitis can affect the development of hordeolum or chalazion, but it is mainly limited to infectious conditions. Herein, we focused on pediatric and adolescent patients with relatively idiopathic allergic conditions rather than infectious or other inflammatory conditions of the eyelid. Thus, we may need to look at this in terms of the characteristic traits of the population. The combination of an immunopathological system, in which activation of mast cells, cytokines, and disruption of the conjunctival epithelial cell junction are excessive^8^, and frequent rubbing, which accelerates the immunologic process, can promote a second flush of eyelid inflammation, even if the allergic condition is not contagious.

However, there was no significant difference in likelihood of undergoing incision surgery between age groups. This can be due to biological and environmental factors. Given the typical progression of allergic flair in children^9^, there is a drastic increase in allergic rhinitis, which is commonly associated with ocular symptoms of AC during the late phase of childhood^10,11^. Beyond the expectation that the likelihood of undergoing incision surgery would be higher during the late phase of childhood, there was no significant difference between the 3–5, 10–12, and 13–19 years age groups. This results can be explained by environmental factors such as regional severity of fine dust or the gradual decrease in the prevalence of allergic diseases^12^. Against this background, a higher risk of undergoing incision surgery accompanied by AC has become a disease that should be noted and looked for throughout the pediatric and adolescent age. In addition, we found that the incision surgery was high when steroids that indirectly reflected severe AC were used. We, therefore, suggest that the development of chalazion and hordeolum, and thus the incision surgery for these eyelid disorders can be considered as a complication following AC.

This study has several limitations. First, there is a possibility that some cases were incorrectly diagnosed as AC in certain busy clinical situations. To avoid such misdiagnosis and overestimation, we excluded conditions that affected eyelid inflammation other than AC and provided a fine definition of inclusion with a combination of diagnosis codes and accompanying allergic eye drops. In this process, however, children who were not prescribed allergic eye drops might have been excluded even if they had AC. Whereas, some patients might not seek medical help, which could lead to underestimation of the prevalence of AC. This may be balanced by some incorrect cases of AC, which might have been overestimated. Additionally, since this is a sample cohort data, it may not completely reflect data from the entire population. Second, the follow-up period was short; thus, the number of incident cases of incision surgery was not high. Herein, we focused on the short-term effect of AC in the current study, and sufficiently significant results based on the cohort study design were obtained despite the short follow-up period. Third, other than age and sex, potential confounders could not be considered in the statistical analysis. Additionally, the clinical severity of AC could not be determined from the HIRA database. To avoid confounding factors as much as possible, we attempted to exclude various conditions that are known to affect the development of hordeolum and chalazion to assess the sole effect of AC on incision surgery. Based on the results, AC can be considered as one of the factors significantly influencing incision surgery and can be incidentally explained in clinical practice. Fourth, we analyzed steroid use as a severe AC. However, we could not evaluate the duration or continuity of drug use for the severity of AC. Additionally, we did not obtain data on hospitalization in our study. Further studies are required to assess this hypothesis. The strengths of our population-based cohort study were its large sample size, strictly defined highly reliable diagnostic criteria, and provision of notable evidence for quantified risk of undergoing incision surgery in pediatric and adolescent patients with AC.

In conclusion, the likelihood of undergoing incision surgery within a year in the pediatric and adolescent population with AC was four times higher than that of the non-AC participants. Our study provides valuable information to help clinicians provide a proper warning to caregivers of pediatric and adolescent patients, such as early management of AC in the pre-allergic season and prohibiting eye rubbing, to avoid possible incision surgery as a complication of AC. Furthermore, a well-controlled cohort study with a longer follow-up period is needed to verify this finding.

## Data Availability

The raw data used in this study can be requested from any qualified investigator through the national HIRA system.
